# Acute Calcific Tendinitis of the Longus Colli Muscles: An Entity That Should Be Known by Emergency Radiologists

**DOI:** 10.7759/cureus.25518

**Published:** 2022-05-31

**Authors:** Pir Abdul Ahad Aziz Qureshi, Gunnar Bollason, Kjartan Logi Ágústsson

**Affiliations:** 1 Department of Radiology, Landspítali - The National University Hospital of Iceland, Reykjavík, ISL

**Keywords:** longus colli tendinitis, longus colli, acute calcific tendonitis of the longus colli, neck pain, odynophagia, inflammatory neck pain, acute calcific prevertebral tendinitis, retropharyngeal tendinitis, acute calcific retropharyngeal tendinitis

## Abstract

Acute calcific tendinitis of the longus colli muscle (LCM) also called acute calcific prevertebral tendinitis or retropharyngeal tendinitis is an inflammatory process of the LCM that results in acute and debilitating symptoms. Although the imaging appearances of this uncommon condition are specific, due to the rarity of this entity and lack of familiarity, it can be sometimes misdiagnosed as a retropharyngeal abscess. This case report presents characteristic radiological features of the acute calcific tendinitis of the LCM, which may be helpful for the emergency radiologist to accurately diagnose this condition to avoid unnecessary surgical interventions.

## Introduction

Acute calcific tendinitis of the longus colli muscle is a rare self-limiting benign disease process caused by the deposition of calcium hydroxyapatite crystals in the longus colli muscle (LCM). LCM is a paired prevertebral muscle that extends from the first cervical vertebra (atlas) to the third thoracic vertebra along the anterior surface of the vertebral bodies [1-3]. Clinically, these patients present with neck stiffness, neck pain, difficulty in swallowing, limited neck rotational movement, trismus, and low-grade pyrexia [1,4] and can be mistaken for severe diseases such as retropharyngeal abscess, cervical tumor, trauma, or disk herniation [4]. This case report describes the characteristic computed tomography (CT) scan features of acute calcific tendinitis of LCM. Furthermore, it makes the emergency physicians and radiologists aware of this uncommon entity to prevent the diagnostic dilemma, which may lead to unnecessary surgical interventions.

## Case presentation

**Figure 1:**
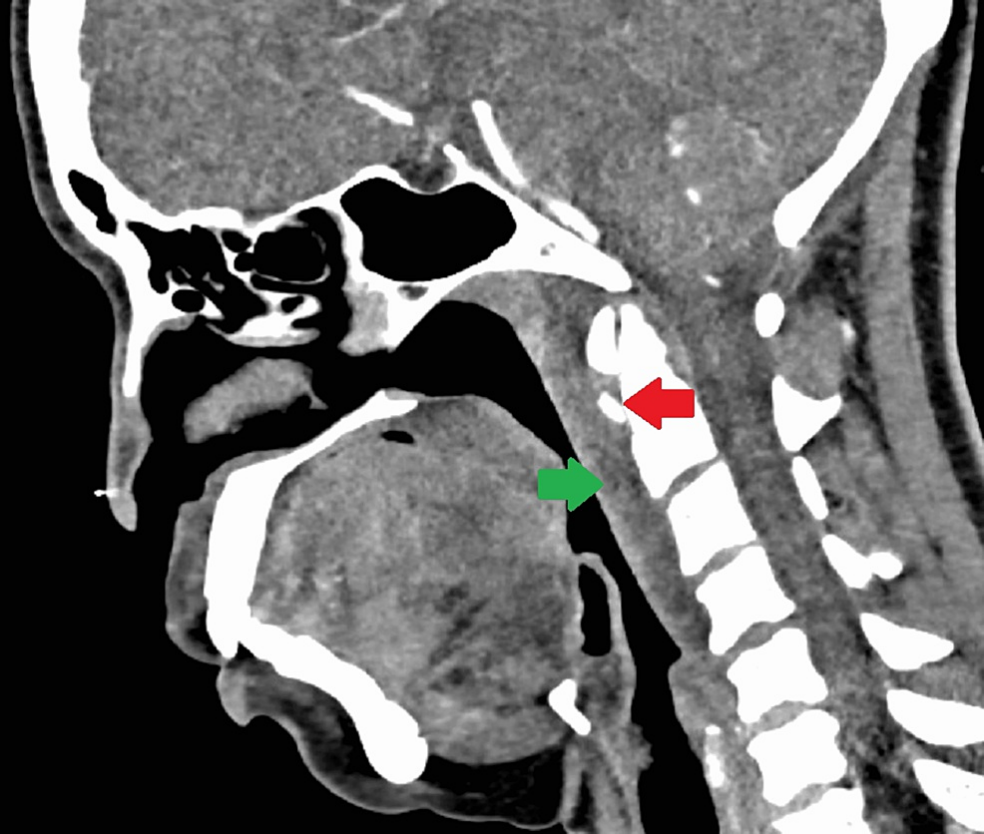
Contrast-enhanced computed tomography (CT) of the neck (sagittal view) Amorphous calcification is seen anterior to the dens (red arrow) associated with prevertebral effusion (green arrow).

**Figure 2:**
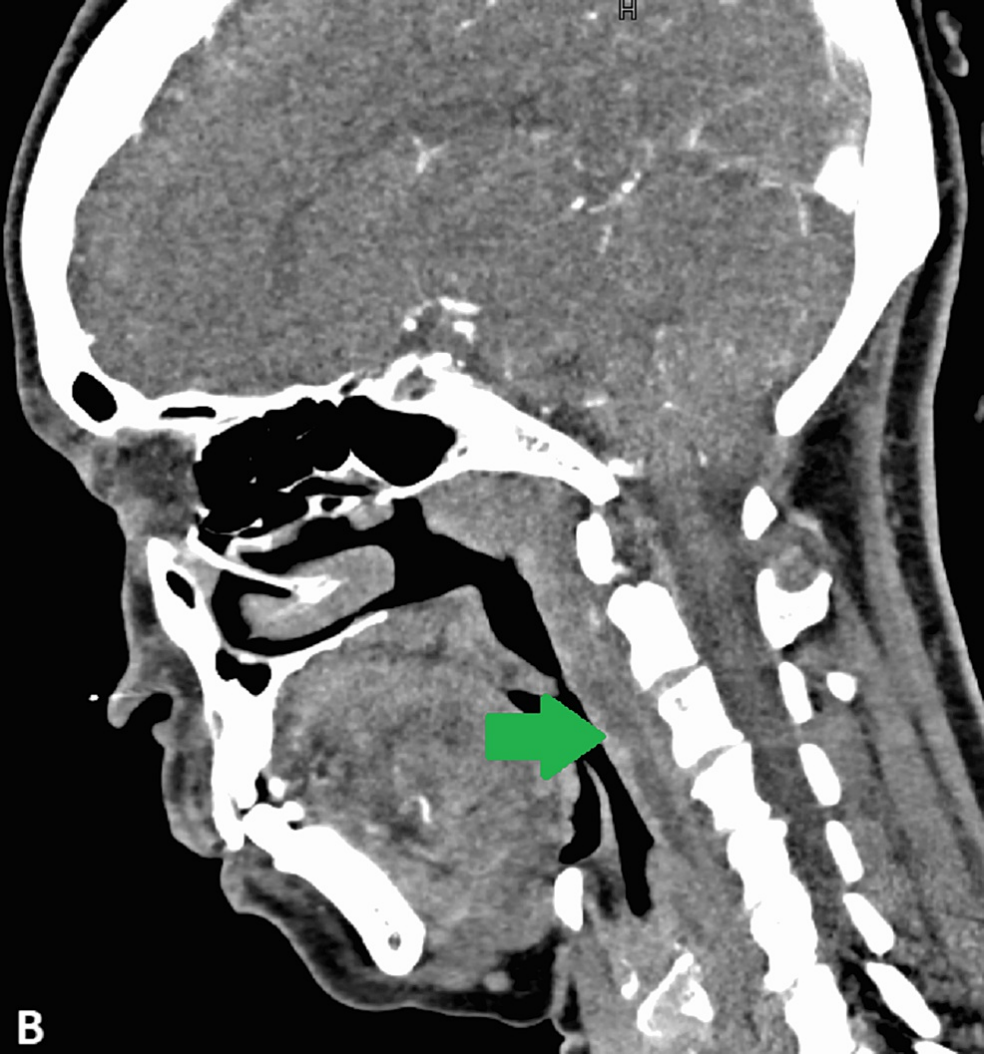
Contrast-enhanced computed tomography (CT) of the neck (sagittal view) Prevertebral effusion can be seen extending down up to the C4 vertebral body (green arrow).

A 48-year-old female presented to the emergency department of our hospital with complaints of neck stiffness, difficulty in opening the mouth, and odynophagia. On general physical examination, she was afebrile and had trismus with restricted movement around the neck. No focal neurological deficit was present. The oropharyngeal and nasopharyngeal mucosa were normal. No mass or lymphadenopathy was present in the neck bilaterally. In addition, the complete blood count (CBC) report was within the normal range. The total leukocyte count was 5.1 x 10^9^/L^ ^(normal range = 4.0-10.5 x 10^9^/L), and C-reactive protein (CRP) was <3 mg/L (normal range ≤ 10 mg/L). She subsequently underwent a CT examination of the neck with contrast to evaluate the cause of pain, revealing 1 cm amorphous calcification anterior to the dens (Figure 1) and retropharyngeal effusion, which was extending up to the C4 vertebral body (Figure 2).

Based on the acute presentation, laboratory, and CT findings, the diagnosis of acute calcific tendinitis of the LCM was made. The patient was then given symptomatic treatment with non-steroidal anti-inflammatory drugs (NSAIDs) and was called for follow-up. The patient is currently feeling better on follow-up, and the pain has significantly decreased.

## Discussion

Acute retropharyngeal calcific tendinitis of the LCM is a self-limiting reactive inflammatory process secondary to the deposition of acute or subacute amorphous calcium hydroxyapatite crystals in the superior tendon fibers of the LCM [4,5], most commonly anterior to the C1 and C2 vertebral bodies [6]. The LCM is paired prevertebral muscles that extend from the C1 to T3 vertebral bodies [4,6] and function as weak neck flexors [6]. The LCM is particularly important in relation to the head and neck as well as spinal pathologies due to their extensive attachment to the cervicothoracic spine and proximity to the retropharyngeal space [6].

The acute retropharyngeal calcific tendinitis of the LCM was first described in 1964 by Hartley. It most commonly occurs between the third and sixth decades without any gender predilection [7]. Hartley described it as an acute case of neck pain and stiffness associated with painful swallowing, which was investigated on plain x-ray that revealed prevertebral soft tissue thickening associated with amorphous calcification anterior to the axis (C2) [6]. In 1994, Ring et al. described five cases of acute retropharyngeal calcific tendinitis of the LCM, which were initially misdiagnosed and led to avoidable medical treatment and even an open biopsy in one patient [4,8]. In a literature review by Park et al., neck pain was the most common symptom in patients with acute calcific tendinitis of the LCM which constitutes about 94%; restricted neck movement and odynophagia were present in 45% of the patients, neck stiffness in 42%, and dysphagia and neck spasm in 27% and 11% of patients, respectively [9].

The specific etiology of this condition is unknown, but possible risk factors include chronic trauma, inflammation, and degenerative changes [1]. Additionally, some authors postulate that this disease can be linked with collagen vascular disorder, renal failure, and osteoarthritis [4]. The degree of calcification can be variable and does not appear to be linked with the severity of symptoms [6].

Clinically, these patients can also present with mild fever and normal or slightly elevated inflammatory markers [1]. Radiologically, on plain x-ray films, the acute calcific tendinitis of LCM typically appears as prevertebral soft tissue thickening with calcification anterior to the C1-C3 vertebral bodies; but some authors have also reported that the calcification may not be evident or missed in the early plain x-ray films, and they can be slightly off the midline and can occur as low as C4-C5 [1]. CT scan is the gold standard imaging modality for the diagnosis of acute calcific tendinitis of the LCM due to its ability to show calcification clearly and to detect prevertebral edema, whereas MRI is excellent in detecting prevertebral edema and effusions but not as sensitive as CT scan in the evaluation of calcification [4,6]. In some cases, MRI also reveals marrow edema in adjacent vertebrae [1]. Bone scintigraphy with technetium 99m-methyl diphosphonate has not been helpful in this condition [6,8].

Additionally, to correctly diagnose the acute calcific tendinitis of LCM, knowledge of the normal anatomy of the cervicothoracic prevertebral muscles and awareness and recognition of the characteristic radiological features are essential [6] to avoid unnecessary medical and surgical interventions as it can be mistaken for multiple other diseases like retropharyngeal abscess, myositis ossificans, fracture-dislocation of the cervical spine, and primary or metastatic neoplasia [6]. Multiple features aid in the correct diagnosis, for example, pathognomonic calcification in the superior fibers of LCM, retropharyngeal fluid without post-contrast wall enhancement, absence of pathological lymph nodes, and absence of adjacent osseous destruction [6]. Acute calcific tendinitis of the LCM is a self-limiting disease that resolves spontaneously within one to two weeks and is usually managed conservatively with NSAIDs and supportive care [1,4].

## Conclusions

Acute calcific tendinitis of the LCM is an uncommon entity that can be misdiagnosed with multiple different pathologies of serious nature ranging from acute infection to the neoplasm. Therefore, knowledge of this entity and awareness of its radiological appearances, especially on the CT scan, are paramount in making the correct diagnosis, especially in the emergency setup, which is essential in preventing unnecessary medical and surgical interventions.
